# Integrated single-cell sequencing, spatial transcriptome sequencing and bulk RNA sequencing highlights the molecular characteristics of parthanatos in gastric cancer

**DOI:** 10.18632/aging.205658

**Published:** 2024-03-18

**Authors:** Xiuli Qiao, Jiaao Sun, Pingping Ren, Hui Guo, Hua Xu, Chongchan Bao, Chunmeng Jiang

**Affiliations:** 1 Department of Gastroenterology, The Second Affiliated Hospital of Dalian Medical University, Dalian, China; 2 The First Affiliated Hospital of Dalian Medical University, Dalian, China; 3 Department of Breast and Thyroid Surgery, Affiliated Hospital of Youjiang Medical University for Nationalities, Baise, Guangxi, China; 4 Key Laboratory of Molecular Pathology in Tumors of Guangxi, Affiliated Hospital of Youjiang Medical University for Nationalities, Baise, Guangxi, China

**Keywords:** gastric cancer, parthanatos, tumor immune microenvironment, single-cell sequencing, spatial transcriptome sequencing

## Abstract

Background: Parthanatos is a novel programmatic form of cell death based on DNA damage and PARP-1 dependency. Nevertheless, its specific role in the context of gastric cancer (GC) remains uncertain.

Methods: In this study, we integrated multi-omics algorithms to investigate the molecular characteristics of parthanatos in GC. A series of bioinformatics algorithms were utilized to explore clinical heterogeneity of GC and further predict the clinical outcomes.

Results: Firstly, we conducted a comprehensive analysis of the omics features of parthanatos in various human tumors, including genomic mutations, transcriptome expression, and prognostic relevance. We successfully identified 7 cell types within the GC microenvironment: myeloid cell, epithelial cell, T cell, stromal cell, proliferative cell, B cell, and NK cell. When compared to adjacent non-tumor tissues, single-cell sequencing results from GC tissues revealed elevated scores for the parthanatos pathway across multiple cell types. Spatial transcriptomics, for the first time, unveiled the spatial distribution characteristics of parthanatos signaling. GC patients with different parthanatos signals often exhibited distinct immune microenvironment and metabolic reprogramming features, leading to different clinical outcomes. The integration of parthanatos signaling and clinical indicators enabled the creation of novel survival curves that accurately assess patients’ survival times and statuses.

Conclusions: In this study, the molecular characteristics of parthanatos’ unicellular and spatial transcriptomics in GC were revealed for the first time. Our model based on parthanatos signals can be used to distinguish individual heterogeneity and predict clinical outcomes in patients with GC.

## INTRODUCTION

Gastric cancer (GC) is the fifth most common cancer worldwide, following lung cancer, breast cancer, colorectal cancer, and prostate cancer [1]. It imposes a considerable health burden, particularly in East Asia. Among the histological types of GC, gastric adenocarcinoma (STAD) is the most common type, accounting for approximately 90-95% of GC cases [2, 3]. Currently, curative surgical resection and adjuvant drug therapy are the standard treatment methods for GC. According to statistics, with the development of targeted drugs, endoscopy and surgery, the global incidence and mortality of GC have decreased year by year, but the prevalence of GC in East Asia is still very high, accounting for more than 70% of the new diagnoses and deaths of GC in the world [4, 5], and it’s worth noting that the prevalence of GC in people under 50 years old has increased year by year globally [4]. This may be linked to genetics, obesity and dysregulation of the microbiome [6]. Additionally, GC is a highly heterogeneous disease in terms of clinical phenotype and molecular patterns. The heterogeneity of the tumor microenvironment and interaction with the host can potentially influence the disease progression, making the prognosis prediction for GC patients very challenging [7, 8]. Therefore, it is crucial to urgently identify sensitive and effective methods to evaluate clinical data in GC patients in order to optimize their treatment process and prognosis.

In 2007, Ted Dawson discovered a novel form of programmed cell death that is dependent on DNA damage and PARP-1 activation, and named it parthanatos [9]. Subsequent research has increasingly confirmed that the parthanatos pathway is widely involved in the occurrence and development of various diseases, including Parkinson’s disease, diabetes, heart failure, cerebral ischemia-reperfusion injury, and others [10]. PARP-1 (Poly (ADP-Ribose) Polymerase 1) is a DNA repair enzyme that mainly exists in the nucleus of eukaryotic cells, accounting for over 90% of cellular PARP. Under normal physiological conditions, PARP-1 monitors the DNA replication process, identifies and approaches DNA damage sites, promotes the recruitment of DNA repair effector proteins, and plays a role in repairing DNA damage. However, under pathological conditions with substantial DNA damage, PARP-1 is excessively activated, catalyzing the breakdown of intracellular nicotinamide adenine dinucleotide (NAD) into nicotinamide and poly ADP-ribose (PAR), resulting in significant depletion of NAD and accumulation of PAR. PAR then migrates to the mitochondria, leading to inhibition of the tricarboxylic acid cycle, impaired mitochondrial energy metabolism, release of apoptosis-inducing factor (AIF) and migration inhibitory factor (MIF) to the nucleus, chromatin condensation and degradation, ultimately resulting in parthanatos [11, 12]. In the development and progression of various cancers, parthanatos has demonstrated elevated activity [13]. The expression levels of PARP-1 and associated genes are generally higher in various cancers, such as breast cancer, ovarian cancer, endometrial cancer, lung cancer, and prostate cancer, when compared to normal tissues [14]. Furthermore, studies have shown that mice with PARP-1 gene knockout exhibit varying degrees of inhibition in tumorigenesis, particularly in pancreatic cancer and colorectal cancer [13, 15]. In GC, the upregulation of PARP-1 expression has been shown to be associated with poor prognosis [16], however, due to the complex cascade reactions and involvement of multiple signaling factors in parthanatos, the specific mechanism of the parthanatos pathway in the occurrence and development of GC has not been elucidated. Therefore, our research will start from here to explore the potential link between parthanatos and GC, aiming to provide scientific guidance strategies for the clinical treatment of GC.

In this study, we first selected parthanatos-related genes from the GeneCards database. Based on TCGA and GEO databases, we collected multi-omics data of parthanatos-related genes in various human cancers. The analysis of integrated single-cell and spatial transcriptome data contributes to the understanding of the structure of cell type distribution and the cellular communication mechanisms that underpin this structure. We introduce this section to explore the differences in the expression of parthanatos signal between GC tissues and normal tissues. Subsequently, we established a GC patient classification model based on parthanatos-related gene expression patterns based on unsupervised cluster analysis. This model can distinguish GC patients based on their different parthanatos characteristics. Furthermore, we used the differentially expressed genes between subtypes to develop a specific parthanatos-related prognostic model for GC, and co-predicted the clinical outcome of patients together with the clinical characteristics of patients. In summary, through a series of bioinformatics analyses, we not only explored the single cell and spatial transcriptomic molecular characterization of parthanatos in GC, but also provided personalized guidance for the clinic treatment and prognosis of GC patients.

## MATERIALS AND METHODS

### Sample source and parthanatos-related gene source

Based on the TCGA platform, we downloaded and curated multi-omics data of pan-cancer in humans, including CNV, SNV, and methylation data, as well as mRNA expression profiles and corresponding clinical information at the transcriptome level. The processing methods for these data were similar to previous studies [17, 18]. Visualization of the results was achieved using the R language and TBtools software. In addition to the pan-cancer data, we focused on in-depth analysis and exploration of the transcriptome data of GC. Specifically, we downloaded the corresponding data from the TCGA-STAD cohort, which included a total of 407 samples, including 32 adjacent non-cancer samples and 375 GC samples. After excluding samples with a survival time of less than 30 days, we analyzed the remaining 312 GC samples with complete follow-up information. Furthermore, to obtain more accurate and convincing results, we collected additional publicly available GC transcriptomic data (GSE84437). After integrating the prognostic information and expression profiles, we obtained a total of 431 GC samples with complete follow-up information. Since different data sources may have batch effects, we performed batch correction using the methods from previous studies [19, 20]. GeneCards is a comprehensive online gene information database that provides detailed information about human genes, including gene function, expression, disease associations, mutations, protein information, and drug association [21]. Users can easily search and browse thousands of genes to gain a deeper understanding of their important roles in biology, medicine, and drug development. All parthanatos-related genes in this study were obtained from the GeneCards platform, where we retrieved 32 parthanatos-related genes for subsequent in-depth analysis.

### Analysis of single-cell sequencing and spatial transcriptome data

The single-cell GC dataset is derived from GSE163558, which includes 3 GC tumor samples (GSM5004180, GSM5004181, and GSM5004182), 1 tumor-adjacent sample (GSM5004183) and 6 metastasis sample (GSM5004184, GSM5004185, GSM5004186, GSM5004187, GSM5004188, GSM5004189) [22]. In order to perform quality control on our raw count data, the following criteria were set: A) min.cells = 3, min.features = 200, B) nCount_RNA >= 1000, C) 200 <= nFeature_RNA <= 8000, D) percent.mt <= 20. To address the issue of differing total counts among cells, we applied a global scaling normalization method (LogNormalize) to normalize the data. This involved normalizing the feature expression measurements of each cell by their total expression, followed by multiplication by a scaling factor (10000), and finally transforming the values using the natural logarithm. Based on the “vst” algorithm, we identified feature genes that exhibited high intercellular differences in the dataset and normalized all genes for further analysis.

To simplify computations and remove data noise, we performed a joint analysis of PCA and Harmony, aiming for batch correction and dimensionality reduction. Harmony applies principal component analysis to embed the transcriptomic expression profile into a lower-dimensional space and then iteratively removes dataset-specific effects. Based on the study of Jiang et al., we manually annotated the single-cell data after quality control and identified a total of 7 cell types, including myeloid cell, epithelial cell, T cell, stromal cell, proliferative cell, B cell, and NK cell [22]. We visualized the above cell subgroups in the form of UMAP [23]. Additionally, we employed six algorithms to score gene sets in the single-cell dataset: AUCell [24], Ucells [25], Singscore [26], ssGSEA [27], addmodulescore [28, 29], and scoring [17]. The scoring value is the sum of scores from the previous five methods and serves to assess the overall distribution of parthanatos gene set scores more stably and comprehensively.

The spatial transcriptomics data were obtained from the GSE186290 dataset. It is important to note that this dataset consists of spatial transcriptomic sequencing of tissue samples from GC mice, and there are currently no publicly available human GC spatial transcriptomics data for analysis. The Read10X_Image function was used to read the spatial distribution information of tissue images and cells, while the Load10X_Spatial function integrated the spatial expression profile with spatial localization information. Similar to the single-cell analysis, we used the aforementioned six gene set scoring methods to evaluate the parthanatos scores of each cell. Finally, we used the SpatialFeaturePlot function to visualize the spatial distribution of parthanatos scores.

### Construction of parthanatos molecular classifier for GC

As research progresses, the scientific community is gradually realizing the significant molecular heterogeneity both between and within tumors. It is due to this heterogeneity that individuals with the same disease often have different treatment response strategies and clinical outcomes. In light of this, we have developed a novel molecular classifier for GC based on parthanatos-related genes. Our goal is to clearly distinguish GC patients with different parthanatos features.

First, we merged the GC transcriptomic data from the TCGA-STAD cohort and the GSE84437 cohort, resulting in a total of 743 GC samples. Molecular clustering analysis was performed using the “ConsensusClusterPlus” R package developed by Wilkerson et al. [30], with the following specific parameters: reps=50, pItem=0.8, clusterAlg=“km”, distance=“euclidean”, maxK=9. The optimal number of clusters was determined based on the consensus cumulative distribution function and delta area plot. The clustering of GC patients was based on unsupervised clustering of parthanatos-related genes. To evaluate the classification performance and clinical relevance of this classifier, we further assessed the parthanatos scores and clinical prognostic differences among different subtypes of GC patients. Additionally, we generated a heatmap to visually depict the expression characteristics of each parthanatos-related gene in this molecular classifier.

### Identification of internal molecular characteristics of the parthanatos molecular classifier

To fully explain the different clinical outcomes among GC patients with different molecular subtypes, we conducted in-depth research and exploration of their intrinsic molecular features. Firstly, based on the KEGG database, we collected 42 classical metabolic pathways and 24 classical immune pathways. The “GSVA” package was used to evaluate the metabolic and immune signaling strength in the 743 GC samples. Finally, we depicted the distribution of the intensity of each metabolic and immune signal in the form of a heatmap. In general, metabolic reprogramming and immune microenvironment are classical molecular markers of tumors, and different forms of metabolic reprogramming and immune microenvironment features may be potential reasons for the different prognostic outcomes of different parthanatos subtypes.

Apart from the immune pathways, there are many other algorithms in bioinformatics that can evaluate the tumor immune microenvironment. Therefore, we have subsequently conducted various immune-related algorithms to explore the inherent relationship between the parthanatos subtype and the tumor immune microenvironment. A) The “Estimate” package is used to predict the content of stromal cells and immune cells in malignant tumor tissues based on gene expression data. This algorithm is based on enrichment analysis of individual sample gene sets and produces four scores: a) stromal score (indicating the presence of stroma in tumor tissue) b) immune score (representing the infiltration of immune cells in tumor tissue) c) ESTIMATE score (stromal score + immune score) d) tumor purity. Using the “Estimate” package, we calculated the aforementioned four scores for 743 GC patients and conducted corresponding comparisons. B) TIMER2.0 online platform provides seven algorithms for predicting immune cell infiltration in tumor samples, including: TIMER, CIBERSOFT, CIBERSOFT-ABS, QUANTISEQ, XCELL, EPIC, and MCPCOUNTER [31]. The specific procedure is as follows: based on R language, the 743 GC samples were divided into 5 groups, with each group containing 150 samples, and the last group containing 143 samples. The expression matrix of these five groups was uploaded to the Immune Estimation module of the TIMER2.0 platform for prediction, and the results of immune cell infiltration were then downloaded and merged. Heatmaps were generated to show the immune cell infiltration of each sample and calculate the corresponding statistical differences [32]. C) Immune checkpoints are the main limiting factors for immune cells to exert anti-tumor functions. Therefore, we collected classical and recognized immune checkpoints from previous literature [33], and compared the expression characteristics of immune checkpoint-related genes in different parthanatos subtypes of patients. D) The association between each parthanatos-related gene and the GC immune microenvironment was calculated. Based on previous literature reports [34], we identified a set of 29 genes related to immune cells and immune-related functions. The ssGSEA algorithm was used to evaluate the immune cell and immune function scores of 743 GC samples, and Spearman correlation analysis was performed to explore the potential association between each parthanatos-related gene and immune cell infiltration and immune function. At the same time, the correlation between parthanatos score and immune cell infiltration and immune function was calculated.

### Identification of potentially sensitive drugs for GC patients based on parthanatos molecular classifier

Danielle Maeser and colleagues developed a novel tumor drug sensitivity prediction scheme, namely “oncoPredict” package [35]. This R package connects *in vitro* and *in vivo* drug screening, allowing easy prediction of tumor response to a large number of drugs screened in cancer cell lines. In this study, we utilized the “oncoPredict” package and the GDSC2 dataset to predict the response of 743 GC patients to each drug and ultimately analyzed potential beneficial drugs for different parthanatos subtypes of GC patients.

### Developing a novel GC prognostic assessment model based on parthanatos molecular classifier

Based on the “limma” package, differential gene expression between different parthanatos subtypes was analyzed. The “clusterProfiler” package was used to assist in Gene Ontology (GO) enrichment analysis and KEGG pathway enrichment analysis [36–38]. Subsequently, the differentially expressed genes were used to construct a GC prognostic model. Firstly, 431 GC samples from the GSE84437 dataset were randomly divided into a 6:4 ratio. 60% of the GC samples were considered as the training cohort for the model (260 GC samples), while the remaining 40% of the GC samples were considered as an internal validation set 1 (test1 cohort, consisting of 171 GC samples). Additionally, all samples from the GSE84437 dataset were treated as an internal validation set 2 (test2 cohort, consisting of 431 GC samples). The GC samples from the TCGA-STAD dataset were considered as the external validation set for the model (test3 cohort, consisting of 312 GC samples).

Firstly, in the training cohort, the model was constructed through the following steps: A) Univariate Cox regression was used to select genes that are related to GC prognosis. B) LASSO regression was applied to filter genes in order to avoid gene collinearity and overfitting of the model [39]. C) Multivariate Cox regression was used to build the prognostic model (based on the predict function to calculate the risk value for each GC patient). D) Patients were divided into high-risk and low-risk groups based on the median risk value. E) The “survival” and “survminer” packages were used to evaluate the modeling effect of the prognostic model by comparing the survival differences between different risk groups of GC patients [40]. F) The “survivalROC” package was utilized to plot the ROC curve and calculate the AUC value to evaluate the predictive accuracy of the model [41]. Subsequently, in the internal validation set 1, internal validation set 2, and external validation set, the same model genes selected from the training cohort were subjected to multivariate Cox regression. The predict function was used to predict the risk value for each sample. Based on the median risk value from the training cohort, all samples from the different datasets were grouped accordingly. Finally, the stability of the model was verified through internal and external validation strategies. To further facilitate the wide application of the prognostic model in clinical settings, clinical features were introduced as variables in the model [42]. Based on the “rms” package, a nomogram was constructed, integrating the patient’s risk group, grade, stage, gender, and age [32]. Calibration plots and ROC curves were used to evaluate the predictive accuracy of the nomogram.

### Differences in immune microenvironment between high and low risk group

To investigate the potential reasons for the different outcomes between high- and low-risk groups, we subsequently conducted immune microenvironment analysis on samples from both groups. Based on the immune-related scores obtained during molecular clustering, we compared the corresponding differences between the high- and low-risk groups. Specifically, we used the TIMER2 platform to predict the immune cell infiltration abundance using seven immune algorithms, and the statistical differences in immune cell infiltration between the high- and low-risk groups were evaluated using the wilcox.test function. Additionally, we also compared the expression of immune checkpoint genes between different risk groups.

### Availability of data and materials

The datasets analyzed in this work may be found in the Supplementary Materials or the first author may be contacted.

## RESULTS

### Pan-cancer analysis based on genomics and transcriptomics

**Figure 1:**
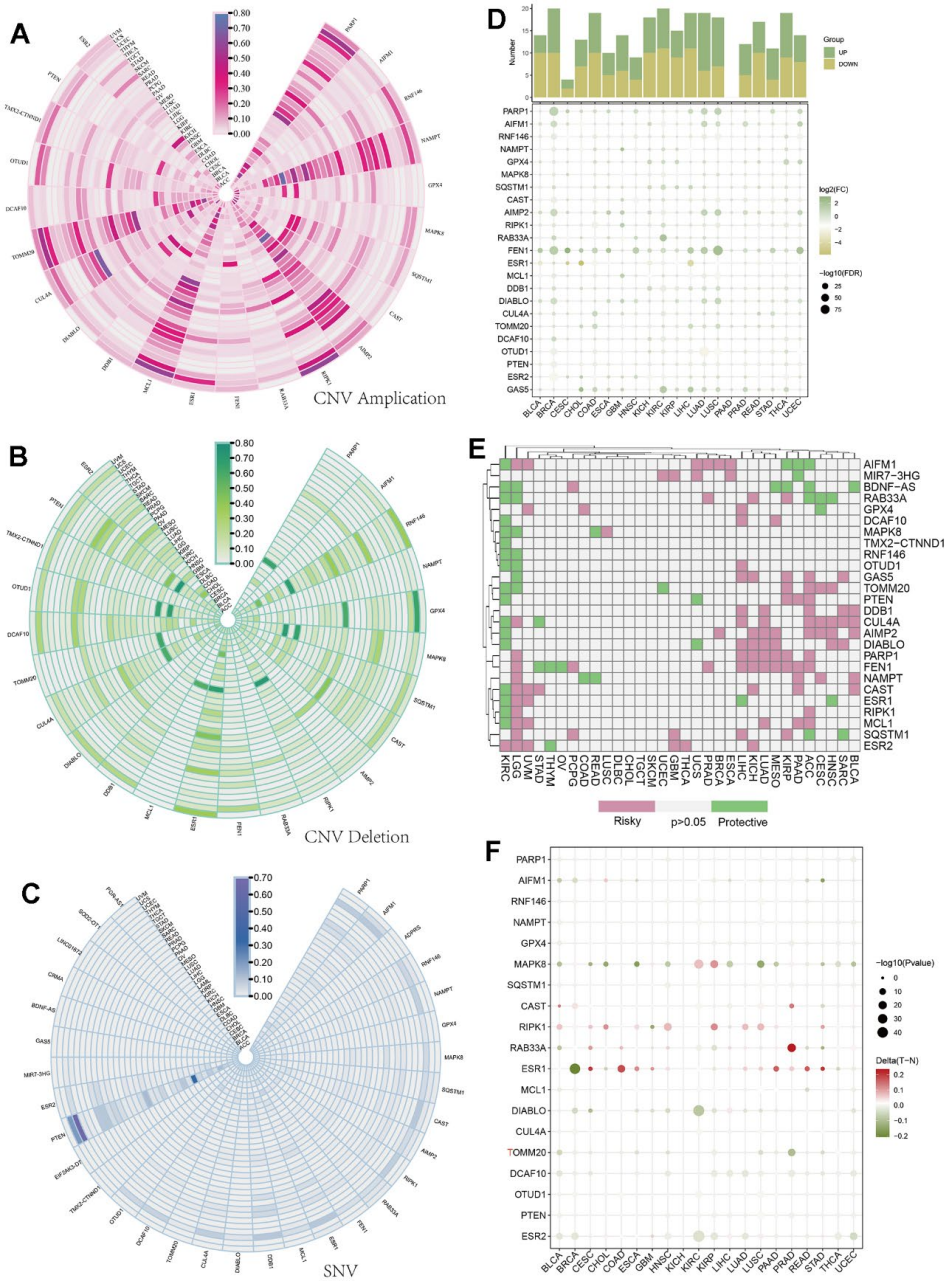
**Pan-cancer analysis of parthanatos-associated genes.** (**A**) CNV amplification data of parthanatos-related genes in different tumor types and the fan color represents the amplification frequency. (**B**) CNV deletion data of parthanatos-related genes in different tumor types and the fan color represents the deletion frequency. (**C**) SNV mutation data of parthanatos-related genes in different tumor types and the fan color represents the frequency of SNV. (**D**) In the expression data of parthanatos-related genes in different tumor types, the color of the squares represents the value of log2 (FC), and the size of the squares represents the value of -log2 (FC). (**E**) The risk profile of parthanatos-related genes in different tumor types, with pink representing risky, green representing protective, and gray representing no statistical difference. (**F**) The comparison of methylation of parthanatos-related genes between different tumor types and normal tissues, the color of the circle represents the methylation difference, and the size of the circle represents the statistical significance.

To study the variations and expression changes of parthanatos-related genes in various human tumors, we determined the mRNA expression as well as the frequency of copy number variations (CNVs) and single nucleotide variations (SNVs) of parthanatos-related genes in different tumors, using sample data from the TCGA database. Genes such as PARP1, NAMPT, AIMP2, MCL1, and TOMM20 exhibited widespread amplifications of CNVs in multiple cancers (Figure 1A), while genes like RNF146, GPX4, ESR1, CUL4A, and PTEN showed widespread deletions (Figure 1B). Figure 1C shows the SNV status of parthanatos-related genes, with genes such as PTEN, DDB1, ESR1, PARP1, and AIFM1 displaying high levels of SNVs in multiple cancers, especially in UCEC, STAD, and SKCM, providing valuable guidance for subsequent experimental research (Figure 1C). In terms of expression, genes like PARP1, FEN1, and GAS5 showed widespread high expression in cancers such as BRCA, LUAD, and LUSC, while genes like PTUD1, ESR1, and ESR2 showed widespread low expression (Figure 1D). Based on survival-related data, we also summarized the risk value of parthanatos-related genes in various cancers (Figure 1E). Additionally, Figure 1F shows the differential methylation levels of parthanatos-related genes between cancer and normal tissues. As shown in the figure, genes like RIPK1, RAB33A, and ESR1 displayed significantly higher methylation levels in cancer tissues compared to normal tissues (Figure 1F).

### Single cell transcriptomic analysis of GC

**Figure 2:**
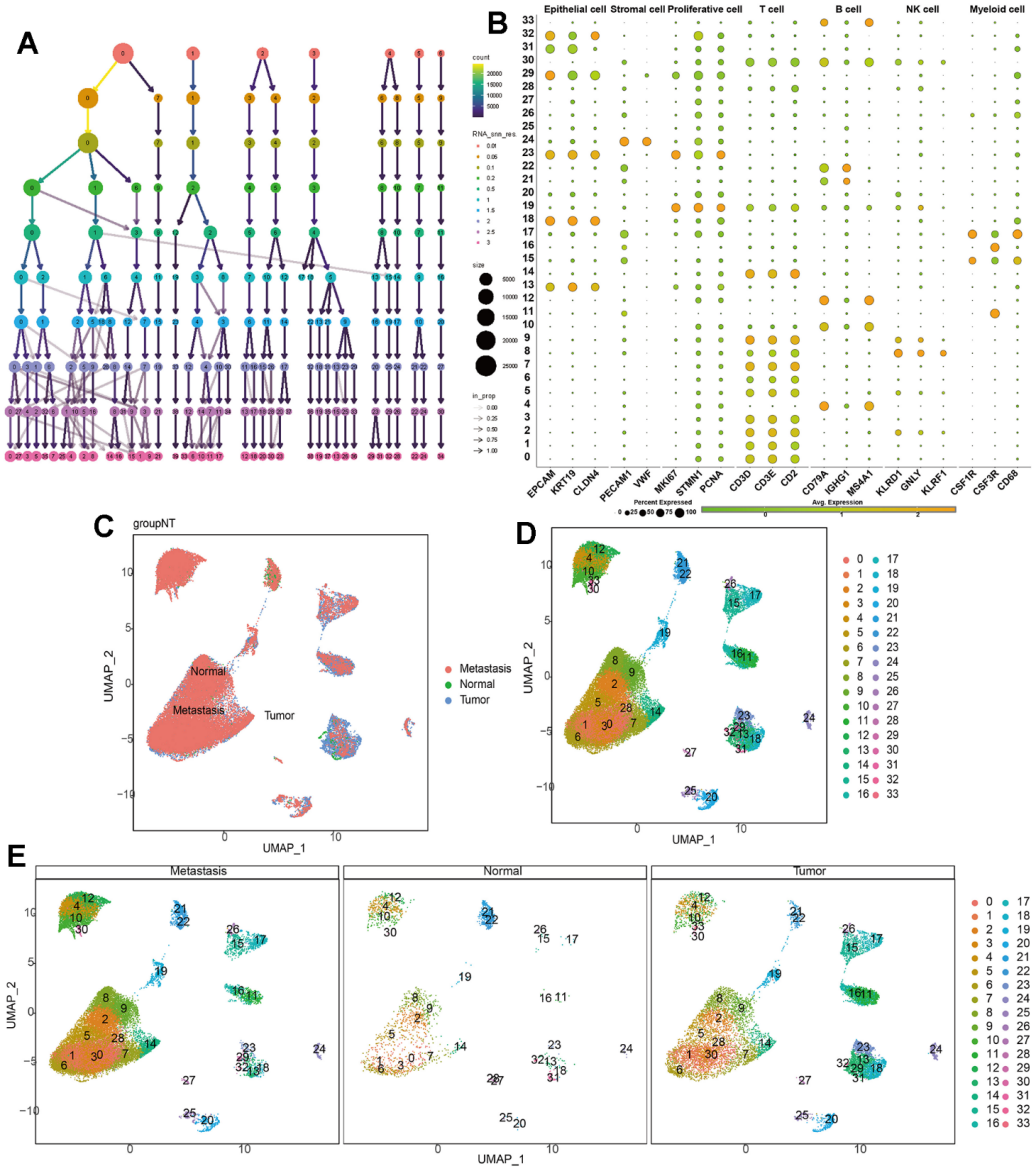
**Single-cell dimensionality reduction.** (**A**) Use the “clustree” package to visualize the division relationships between cell populations under different resolutions. (**B**) Annotate the cell characteristics of 34 clusters. (**C**–**E**) Distribution of 34 kinds of cell clusters in the perspective of UMAP dimensionality reduction algorithm.

**Figure 3:**
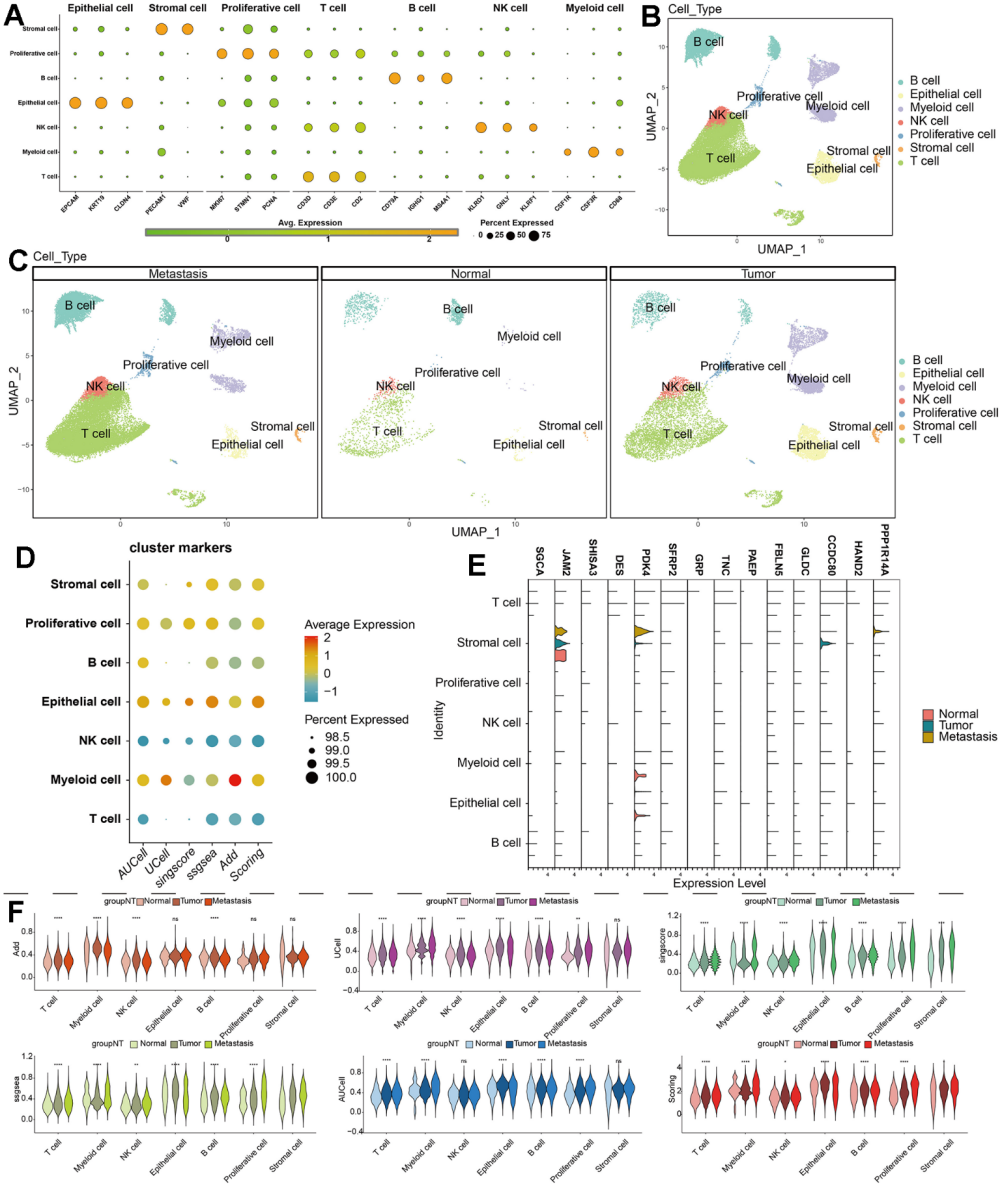
**Single-cell annotation and parthanatos scores prediction.** (**A**) Expression of specific marker genes in 7 cell types. (**B**, **C**) Dimension reduction and annotation of cell clusters based on UMAP algorithm. (**D**) 5 algorithms scored the gene sets of seven cell clusters. (**E**) Comparison of the expression difference of 14 model genes in 7 cell types of normal tissue, tumor tissue and metastatic tissue. (**F**) Comparison of gene set scores of 7 cell clusters among normal tissue, tumor tissue and metastatic tissue.

Our single-cell data consists of 3 GC tumor samples (GSM5004180, GSM5004181, and GSM5004182), 1 tumor-adjacent sample (GSM5004183) and 6 metastasis sample (GSM5004184, GSM5004185, GSM5004186, GSM5004187, GSM5004188, GSM5004189). To perform quality control on our raw count data, we first filtered out unsatisfactory cells based on sequencing depth, number of genes, mitochondrial content, and ribosomal content, there were 53940 cells before quality control, and 41264 cells were left after quality control (Supplementary Figure 1). Subsequently, we conducted a joint analysis of PCA and Harmony to remove batch effects and reduce dimensionality, ultimately obtaining 20 principal components (PCs) for further analysis. During the determination of the resolution value, we observed that increasing the resolution value allowed us to clearly observe which cell clusters were continuously dividing into subclusters, revealing the relationships between cell clusters at different resolutions. When the resolution was set to 2, we observed significant interweaving between cells, so we chose a resolution of 2 and obtained 34 unknown cell clusters (Figure 2A), and the specific maker genes expressed in each cell cluster were shown in bubble charts (Figure 2B). Then we combined all the single-cell samples, and performed dimensionality reduction on the merged samples using tSNE nonlinear clustering algorithms (Supplementary Figure 2), and we displayed 34 unknown cell clusters in the UMAP map, and found that they were expressed and distributed differently in normal, tumor, and metastatic tissues (Figure 2C–2E). Based on cell annotation strategies developed by previous studies, we identified 7 cell types within the GC microenvironment: myeloid cell, epithelial cell, T cell, stromal cell, proliferative cell, B cell, and NK cell, the specific genes expressed by each cell type were shown in the form of bubble maps (Figure 3A), we also labeled the cell clusters on the UMAP map (Figure 3B). Figure 3C depicts the cellular distribution characteristics within normal tissue, tumor tissue, and metastasis tissue.

### Parthanatos gene set scoring based on single-cell and spatial transcriptome data

Cells do not rely solely on one or a few genes to carry out their functions. Many genes in the upstream and downstream pathways of the functional pathways vary in expression as the functions vary in strength. Therefore, we used five algorithms (AUCell, Ucells, Singscore, ssgsea, and addmodulescore) to score the parthanatos gene set in single-cell data. The Scoring score is the total score of the five algorithms. Figure 3D shows the expression of parthanatos-associated genes in 7 cell clusters for each algorithm. Myeloid cell, epithelial cell and proliferative cell show strong signals, while NK cells and T cells show weaker signals (Figure 3D).

**Figure 4:**
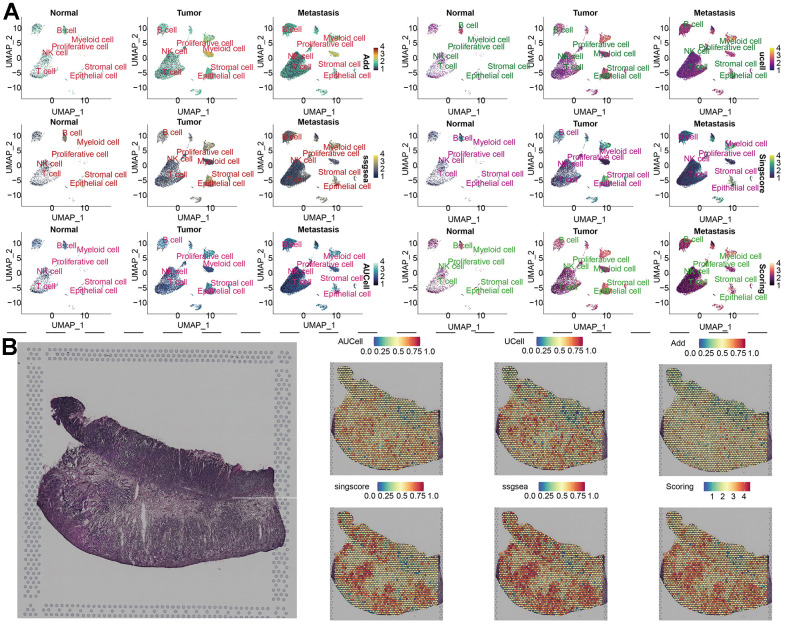
**Distribution of parthanatos scores in single-cell atlas and spatial resolution.** (**A**) Based on the UMAP algorithm, the gene set scores of 7 cell clusters were displayed in the combined samples (including normal tissue, tumor tissue and metastatic tissue). (**B**) Spatial transcriptome data of GC, gene set scoring under 6 gene set scoring algorithms.

To compare the differences in parthanatos-related gene expression among normal tissue, tumor tissue and metastasis tissue, we displayed the gene set scores for each cell cluster under the five algorithms in Figure 3F. The different algorithm scores indicate that compared to normal tissue, almost all cell types within the tumor tissue and metastasis tissue show higher parthanatos gene scores. Notably, the gene set scores for epithelial cell, T cell and proliferative cell in tumor tissue and metastasis tissue are significantly higher (Figure 3F). Furthermore, using the UMAP algorithm, we mapped the 7 cell clusters and their respective parthanatos gene set scores onto the merged sample tissue. By comparing the Scoring scores, we can clearly observe that epithelial cell, T cell and proliferative cell in tumor tissue and metastasis tissue have higher parthanatos gene set scores, which is consistent with our previous observations (Figure 4A). In addition, the control data from mouse GC tissue revealed, for the first time, the spatial distribution of parthanatos signals at the tissue level (Figure 4B).

### Cluster analysis based on parthanatos score

**Figure 5:**
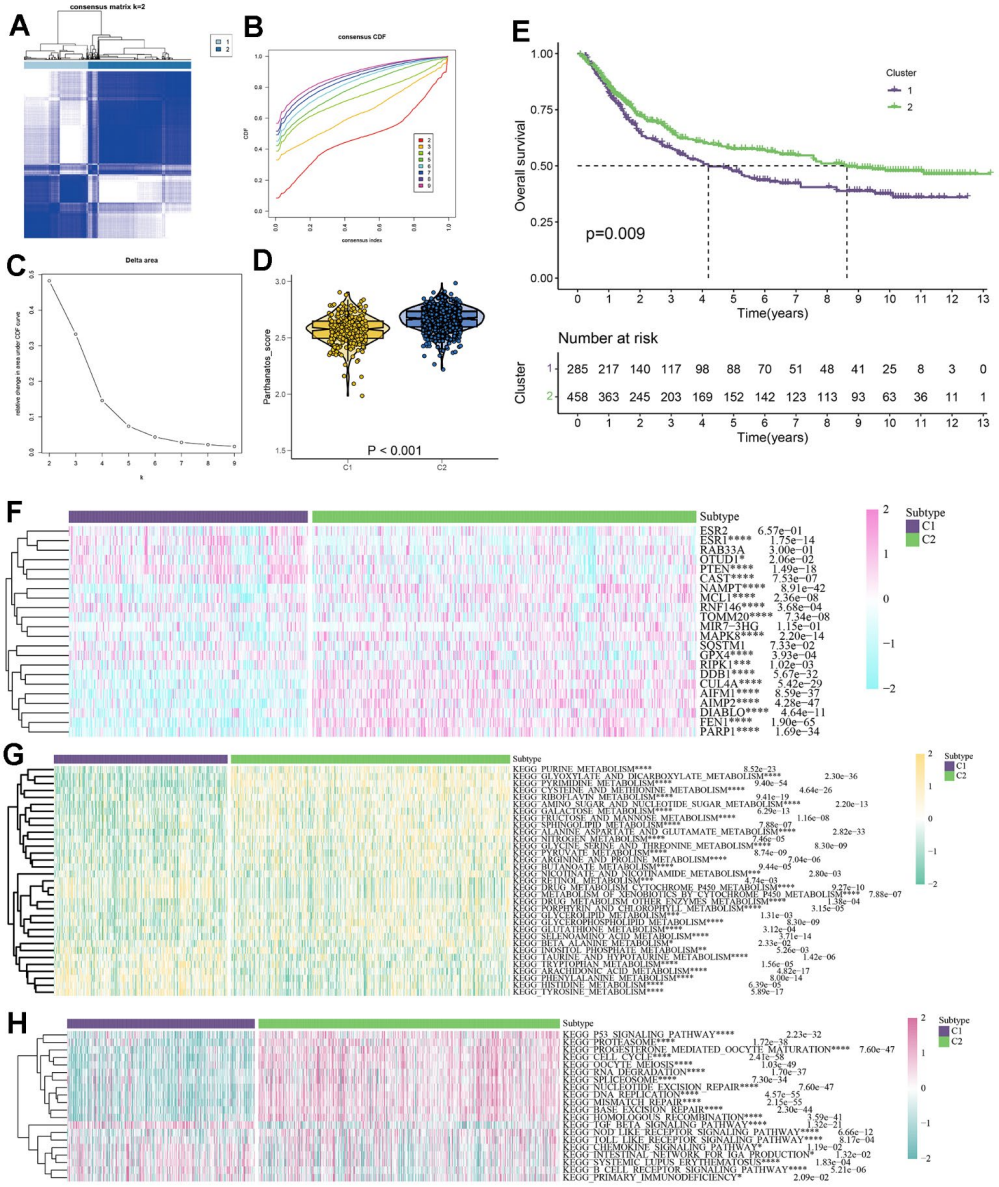
**Parthanatos-based clustering analysis.** (**A**) Define a consensus matrix heat map of k = 2 clusters and their associated regions. (**B**) Cumulative distribution function (CDF) curve under different cluster number. (**C**) The relative change in area under the CDF curve for different values of k. (**D**) The violin plot shows enrichment scores for two clusters (C1 and C2), p<0.001. (**E**) Survival curves of C1 and C2 clusters, purple for C1, green for C2. (**F**) Expression difference of parthanatos-related genes between CI and C2 subtypes. (**G**) Differences in the activity of metabolic pathways between CI and C2 subtypes. (**H**) Differences in immune pathway activity between CI and C2 subtypes.

We developed a GC classification model based on parthanatos-related genes, which can clearly distinguish GC patients with different parthanatos features. Using unsupervised clustering analysis algorithm [43], we divided a total of 743 GC patient samples from TCGA and GEO databases into two subtypes (C1 and C2). The results of consistency matrix heatmap, cumulative distribution curve, and delta area curve all confirmed that k=2 is the optimal clustering number (Figure 5A–5C). Violin plots showed the enrichment scores of the two subtypes, where C2 subtype had higher parthanatos scores, indicating higher activity of parthanatos-related genes, while C1 subtype had the opposite trend (Figure 5D). The heatmap displayed the expression patterns of different parthanatos-related genes between C1 and C2 subtypes. Except for some genes, most genes, such as RIPK1, DDB1, CUL4A, AIMP2, and PARP1, were expressed at higher levels in the C2 subtype compared to the C1 subtype, further confirming the higher expression activity of parthanatos-related genes in C2 subtype patients (Figure 5F). We also studied the prognosis differences between the two subtypes using the “survival” package and “survminer” program in R Studio. It was found that patients with the C2 subtype had better prognosis and higher survival rates, suggesting that a higher parthanatos score is associated with better prognosis in GC patients (Figure 5E).

### Metabolic reprogramming and immune microenvironment characteristics of the parthanatos scoring model

In order to fully explore the intrinsic molecular features of GC patients in different subtypes, we collected 42 classic metabolic pathways and 24 classic immune pathways based on the KEGG database. We evaluated the metabolic and immune signaling intensities in 743 GC samples and depicted the distribution of each metabolic and immune signal in the form of heat maps. In the C1 subtype, signals of metabolic pathways such as retinol metabolism, drug metabolism cytochrome P450 metabolism, and metabolism of xenobiotics by cytochrome P450 metabolism were enhanced, while signals of metabolic pathways such as purine metabolism, pyrimidine metabolism, cysteine and methionine metabolism, and riboflavin metabolism were weakened. The opposite was observed in the C2 subtype (Figure 5G). Regarding immune-related pathways, signals of immune pathways such as TGF beta signaling pathway, chemokine signaling pathway, and intestinal network for IGA production were enhanced in the C1 subtype, while signals of immune pathways such as P53 signaling pathway, proteasome, progesterone mediated oocyte maturation, oocyte meiosis, RNA degradation, spliceosome, and nucleotide excision repair were enhanced in the C2 subtype (Figure 5F).

**Figure 6:**
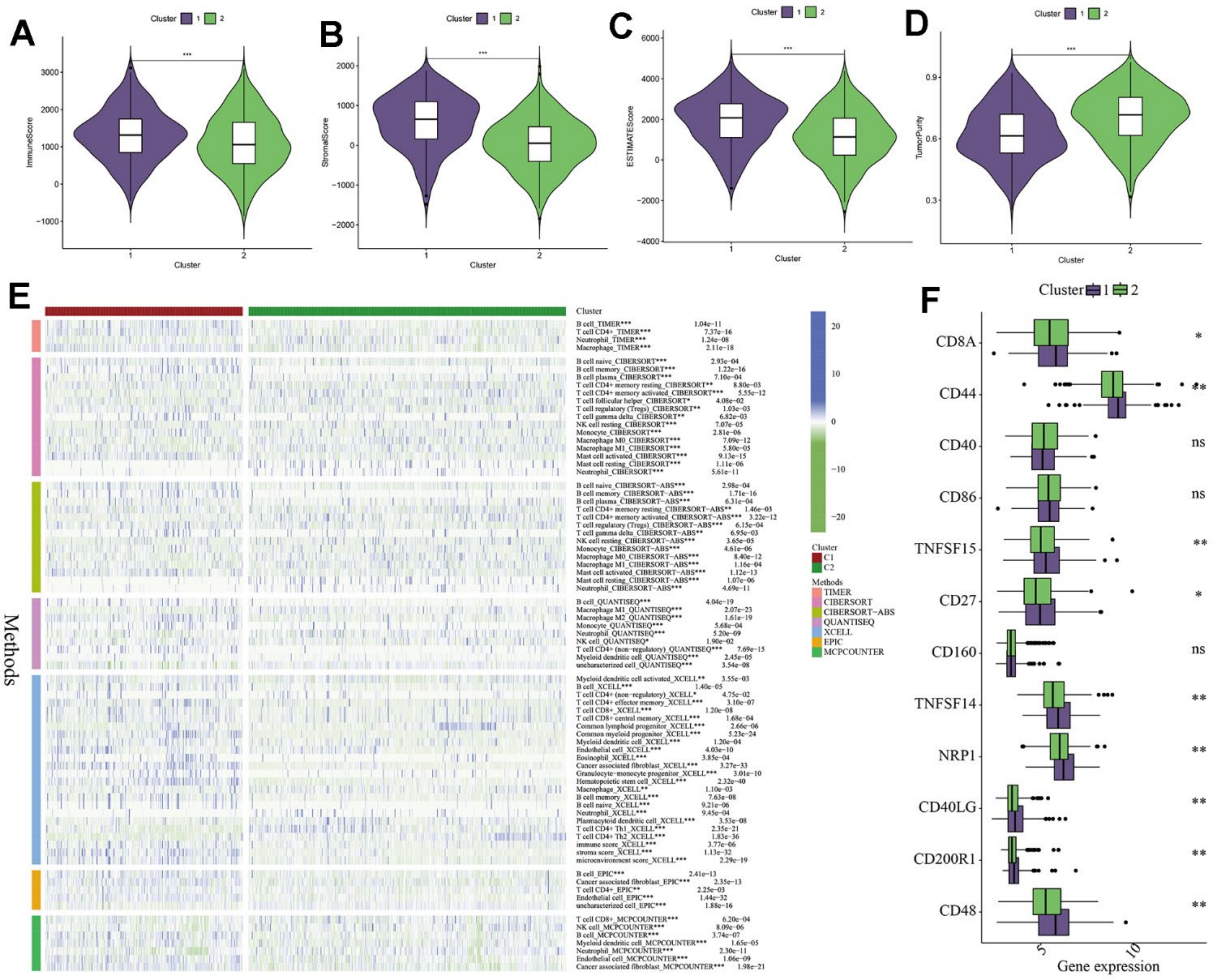
**Analysis of immune microenvironment between different subtypes.** (**A**–**D**) The difference between the two clusters in immune score, stromal score, ESTIMATE score and tumor purity, P-values are represented by *. (**E**) Seven algorithms were used to analyze immune cell or functional differences between CI and C2 subtypes. (**F**) Differences in the expression of immune checkpoint related genes between CI and C2 subtypes, P-values are indicated by *. *: p<0.05, **: p<0.01, ***: p<0.001, “ns” indicates no significant difference.

In addition, we used the ESTIMATE algorithm to analyze the proportions of immune cells, stromal cells, and tumor cells in tumor tissues, and obtained the immune score, stromal score, ESTIMATE score, and tumor purity for both C1 and C2 subtypes. From the graphs, it can be seen that C1 subtype had more stromal and immune cells, while C2 subtype had higher tumor purity (Figure 6A–6D). This phenomenon may be caused by immune function suppression and abnormal accumulation of immune cells in the tumor microenvironment of the C1 subtype. To further analyze the differences in the immune microenvironment between the two subtypes, we used seven immune cell infiltration prediction algorithms provided by the TIMER2.0 online platform (including TIMER, CIBERSOFT, CIBERSOFT-ABS, QUANTISEQ, XCELL, EPIC, and MCPCOUNTER) to analyze the extent of immune cell infiltration between the C1 and C2 subtypes. From the graphs, it can be observed that regardless of the algorithm used, the number of B cells in the C1 subtype was significantly higher than that in the C2 subtype, while the opposite was observed for neutrophils. Other immune cells also showed certain differences between the two subtypes (Figure 6E). Finally, Figure 6F shows the expression patterns of immune checkpoint-related genes in the C1 and C2 subtypes. Genes such as CD8A, CD44, NRP1, TNFSF14, TNFSF15, CD27, and CD48 were significantly higher expressed in the C1 subtype (Figure 6F). This result suggests an excessive activity of immune checkpoints in the tumor microenvironment of the C1 subtype, which may lead to tumor immune escape to a certain extent and subsequently cause excessive accumulation of immune cells. This is consistent with our previous findings on the abnormal accumulation of immune cells in the C1 subtype and the differences in immune microenvironment characteristics between the two subtypes.

### Significance of parthanatos scoring model for GC-targeted drug therapy

To further explore the potential value of parthanatos in the clinical treatment of GC patients, we used the “oncoPredict” package and GDSC2 dataset to predict the response of 743 GC patients to various targeted therapies. We analyzed the potential beneficial drugs for GC patients in the C1 and C2 subtypes and plotted box plots to determine the impact of parthanatos on the sensitivity to 12 common targeted drugs in GC. Patients in the C1 subtype showed higher sensitivity to dasatinib, while patients in the C2 subtype showed higher sensitivity to 11 other drugs such as carmustine, sorafenib, rapamycin, and sepantronium (Supplementary Figure 3A–3L). This result may provide precise guidance for future individualized targeted drug therapy for GC patients.

### Relationship between parthanatos and immune cell infiltration in GC tissues

**Figure 7:**
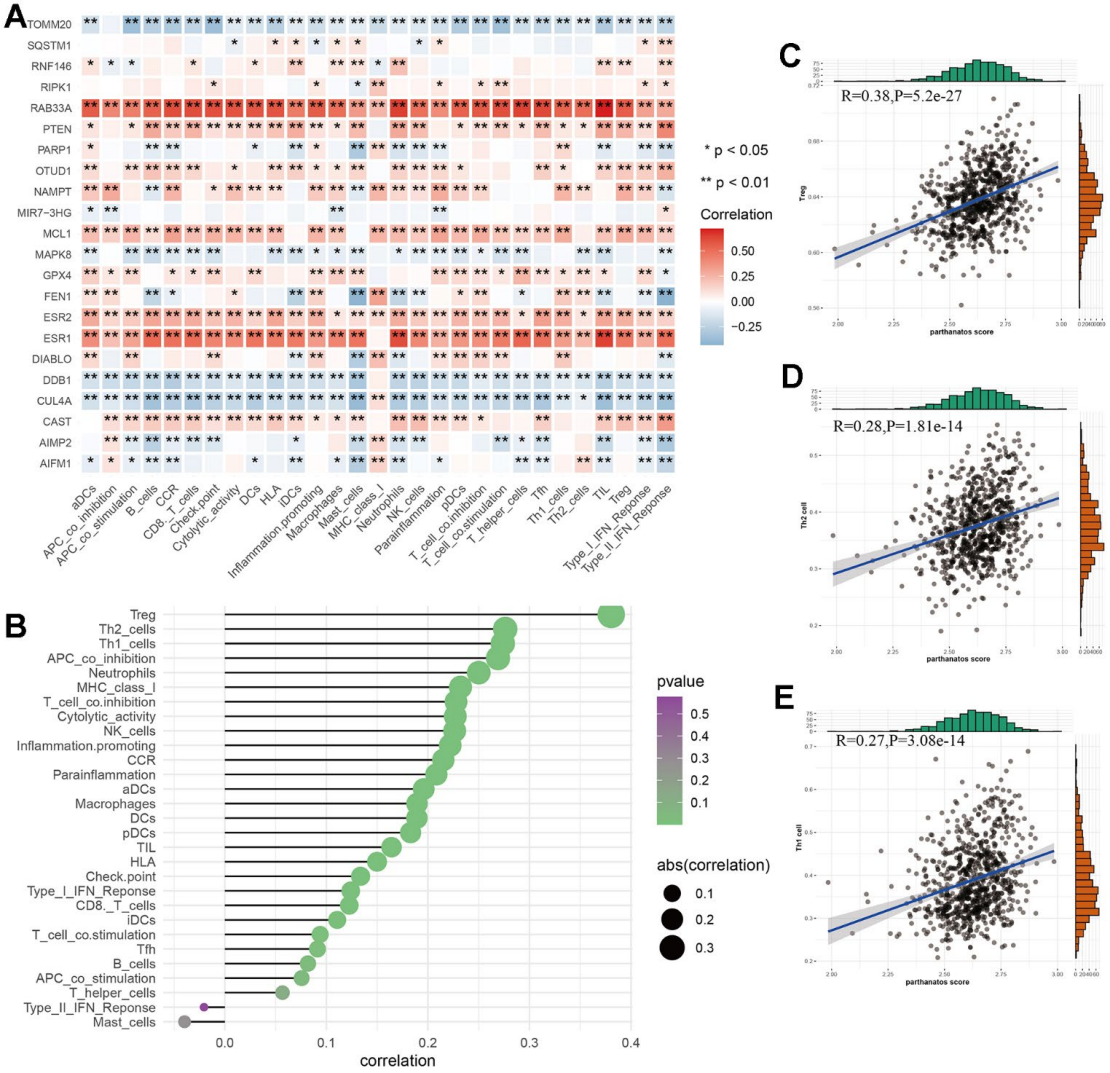
**Relationship between parthanatos score and immune microenvironment in GC.** (**A**) Heat map depicting the relationship between parthanatos-related genes and levels of immune cell infiltration. Positive correlations are shown in red, negative correlations are shown in blue, and p-values are shown by *. (**B**) The bubble plot shows the correlation between parthanatos score and the levels of infiltration of various immune cells, with the size of the circle indicating the size of the correlation and the color of the circle indicating statistical significance. (**C**–**E**) Correlation between parthanatos score and Infiltration levels of three kinds of immune cells. *: p<0.05, **: p<0.01, ***: p<0.001, “ns” indicates no significant difference.

The characteristics of GC include a high degree of heterogeneity in tumor cells and tumor immune microenvironment. To investigate this, we evaluated the immune cells and immune function scores of 743 GC samples based on the ssGSEA algorithm. We explored the potential associations between parthanatos-related genes and 29 types of immune cells or functions using Spearman correlation analysis. We also calculated the correlation between parthanatos scores and immune cell infiltration. We found that different parthanatos-related genes have varying degrees of correlation with various immune cells or functions. Among them, RAB33A, ESR1, and ESR2 were positively correlated with most immune cells or functions, while TOMM20, DDB1, and CUL4A were negatively correlated (Figure 7A). The bubble plot displays the correlation between classic immune infiltration-related cells and parthanatos, including both positive and negative correlations (Figure 7B). We then selected the three immune cells with the highest correlation (Treg, Th2 cell, Th1 cell) and performed correlation analysis with parthanatos scores. The R-values were 0.38, 0.28, and 0.27, respectively, indicating positive correlations (Figure 7C–7E). This finding is consistent with our previous results, where we observed a higher quantity of Th1 and Th2 cells in the C2 subtype compared to the C1 subtype using the XCELL algorithm.

### Construction and verification of survival and prognosis model of GC patients based on parthanatos-related genes

**Figure 8:**
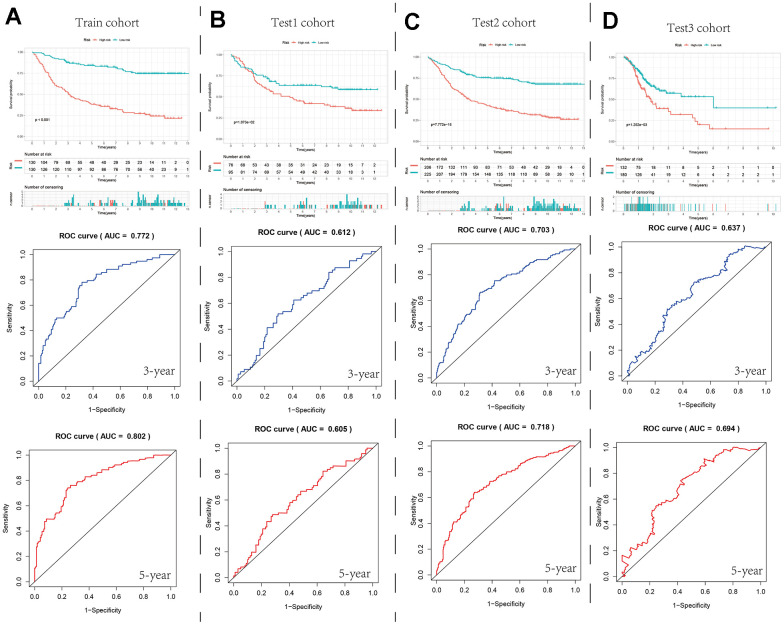
**Survival curves and ROC curves for the high- and low-risk groups in the four cohorts.** (**A**–**D**) Comparison of survival curve and ROC curve between train cohort, test1 cohort, test2 cohort and test3 cohort.

Based on the limma and “clusterProfiler” packages, we screened for differentially expressed genes between different parthanatos subtypes for GO enrichment analysis and KEGG enrichment analysis (Supplementary Figure 4). These genes were then used to construct a prognostic model for GC patients (SGCA, JAM2, SHISA3, DES, PDK4, SFRP2, GRP, TNC, PAEP, FBLN5, GLDC, CCDC80, HAND2, and PPP1R14A), Figure 3E shows the expression of 14 model genes in seven cell types of normal tissue, tumor tissue, and metastatic tissue (Figure 3E). In the train cohort, we performed univariate Cox regression, LASSO regression, and multivariate Cox regression analysis on the differentially expressed genes to build the prognostic model (Supplementary Figure 5). We divided the GC patients in the train cohort into high- and low-risk groups based on the median risk value. We then compared the prognosis differences and analyzed the ROC curve to evaluate the predictive accuracy of the model (Figure 8A and Supplementary Figure 6). Subsequently, in the internal validation set 1 (test1 cohort), internal validation set 2 (test2 cohort), and external validation set (test3 cohort), we performed multivariate Cox regression using the model genes selected from the train cohort. We used the predict function to predict the risk values for each sample and used the median risk value from the train cohort as the cut-off value to divide the three validation sets into high- and low-risk groups. We then conducted prognosis difference comparisons and analyzed the ROC curve. In different cohorts, the high-risk group consistently showed worse prognosis compared to the low-risk group, and the AUC values for 3-year and 5-year ranged from 0.6 to 0.8, indicating the stability and potential generalizability of the prognostic model (Figure 8B–8D and Supplementary Figure 6). In order to explore the differences in the immune microenvironment between the high and low-risk groups, we used the TIMER2 platform to predict the immune cell infiltration abundance using seven immune algorithms in the four cohorts. We also compared the expression differences of immune checkpoint genes between different risk groups (Supplementary Figure 7). The results showed that in all algorithms and cohorts, the low-risk group had higher numbers of plasma cells, NK cells, and certain CD4+ T cells (such as activated memory CD4+ T cells, Th1 cells, and Th2 cells), while tumor-associated fibroblasts (CAFs) showed the opposite trend. Additionally, the box plot results showed higher expression of immune checkpoint-related genes in the high-risk group compared to the low-risk group. These differences in the immune microenvironment may be one of the main intrinsic factors leading to differences in prognosis and clinical data between the high- and low-risk groups.

**Figure 9:**
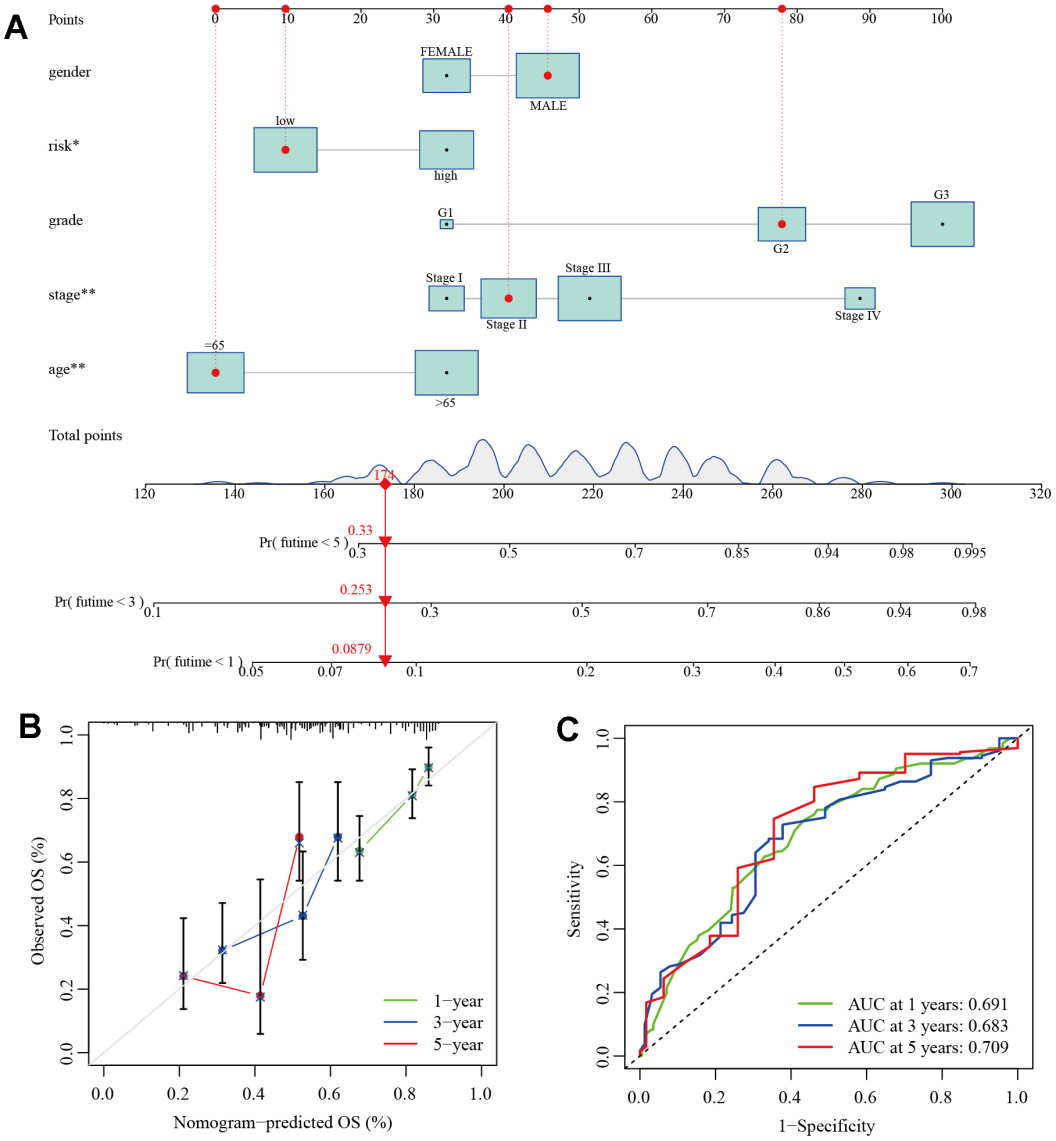
**Establishment of clinical nomogram.** (**A**) A nomogram for predicting prognostic survival time in GC patients. (**B**) Calibration curves of 1-, 3-, and 5-year survival in GC patients. (**C**) ROC curves of 1-, 3-, and 5-year survival in GC patients.

To facilitate the clinical application of the prognostic model, we introduced clinical features of GC as variables in the model and used the “rms” package to construct a column line chart for accurate prediction of GC patient prognosis. The column line chart consists of 10 parallel lines. Each row represents a score, with gender in the second row, risk type in the third row, grade in the fourth row, stage in the fifth row, and age in the sixth row. The total score in the seventh row is obtained by adding the scores of age, grade, stage, and risk type. With this chart, we can easily estimate the survival rates of GC patients at 1, 3, and 5 years (Figure 9A). Furthermore, Calibration plots and ROC curves were used to assess the predictive accuracy of the column line chart. The calibration curves for 1, 3, and 5-year survival closely align with the diagonal line (Figure 9B), and the AUC values were 0.691, 0.683, and 0.709, respectively (Figure 9C). This indicates that both our prognostic model and the column line chart construction are accurate and have predictive value for prognosis.

## DISCUSSION

Unlike other known forms of cell death, parthanatos is a cell death pathway that relies on PARP1 instead of caspase, and its occurrence involves the participation of multiple factors such as NAD and AIF [44]. In the development of cancer, parthanatos interacts closely with other forms of cancer cell death, such as apoptosis and autophagy, playing a significant role in breast cancer, colon cancer, ovarian cancer, and other cancers [10]. As one of the most common malignancies worldwide, GC has been shown to be closely associated with cell death processes such as ferroptosis, pyroptosis, and immunogenic cell death [45–47]. We believe that targeting parthanatos may provide a new approach to precision treatment for cancer. Therefore, this study aims to explore the role of parthanatos in GC and search for new biomarkers for the treatment and prognosis of GC based on the molecular characteristics of parthanatos.

Firstly, we explored the mutation and expression patterns of parthanatos-related genes in various human cancers. We downloaded clinical data from the TCGA database and analyzed the multi-omics data of parthanatos-related genes in different human cancers. Additionally, to determine whether parthanatos could be a potential target for GC treatment, we also analyzed the risk and methylation status of parthanatos-related genes in different types of cancer. Our analysis revealed varying degrees of mutations and expression differences in parthanatos-related genes across multiple cancers, which significantly impacted prognosis risk. These findings open up several potential avenues for future research on parthanatos in human cancers.

ScRNA-Seq is a high-throughput technology that allows for quantitative sequencing of gene expression profiles at the single-cell level, aiding in the deciphering of hidden heterogeneity within cell populations [48]. In a recent study, Shen et al. utilized scRNA-Seq to explore the expression characteristics of mesenchymal stem cells in GC and their role in treatment and prognosis [49]. To investigate the correlation between parthanatos and single cells in GC, we included a dataset from the GEO database (GSE163558) for scRNA-Seq analysis. We identified and classified cells in the samples, resulting in 7 cell clusters expressing cell-specific genes. These clusters include T cells expressing CD3D, CD3E and CD2; NK cells expressing KLRD1, GNLY and KLRF1; stromal cell expressing PECAM1 and VWF; epithelial cells expressing EPCAM, KRT19 and CLDN4; B cells expressing CD79A, IGHG1 and MS4A1; proliferative cells expressing MK167, STMN1 and PCNA; myeloid cells expressing CSF1R, CSF3R and CD68. We further scored the parthanatos gene set for each cell cluster, finding different gene set scores among the clusters, with significant differences between normal, tumor and metastatic tissues. The control data revealed the spatial distribution characteristics of parthanatos in GC tissue for the first time. However, due to the limited availability of control data, statistical analysis could not be conducted. We hope that more control data will become available in the future to further uncover the differences in parthanatos signals between GC tissue and adjacent non-cancerous tissue at the spatial resolution level. Nonetheless, the above discoveries are advantageous in our analysis of tumor heterogeneity in GC at the single-cell level and spatial resolution, providing effective insights into the genetic and functional analysis of GC cells.

The heterogeneity of GC is one of the main reasons why it is challenging to diagnose and treat accurately [50]. To classify GC patients appropriately and explore the role of parthanatos in GC, we merged the gene expression data from the TCGA-STAD cohort and the GSE84437 cohort, resulting in 743 GC samples. Based on the expression levels of parthanatos-related genes, we divided all samples into two subtypes: C1 and C2. C1 had lower parthanatos scores, while C2 had higher parthanatos scores. In previous studies, parthanatos and its related components have been reported to exhibit anti-tumor effects [51, 52], For example, PARP1 negatively regulates epithelial-to-mesenchymal transition (EMT) and inhibits crucial processes such as tumor cell invasion [53]; AIF, as a cell death inducer, prevents the inactivation of the tumor suppressor PTEN by inhibiting its oxidation process, thereby suppressing tumor metastasis [54]. Our survival curve showed that patients in the C2 subtype had better overall survival than those in the C1 subtype, suggesting that protective genes may constitute the majority or dominantly play a role among parthanatos-related genes, which is consistent with previous research findings. On the other hand, our results showed that pathways such as metabolism of xenobiotics by cytochrome P450 were more active in the C1 subtype compared to the C2 subtype. Studies have shown that cytochrome P450 plays a central role in the oxidative activation of various carcinogens and is important in tumor development and response to chemotherapy [55]. We believe that the abnormal activation of certain metabolic pathways may be one of the reasons for the poorer prognosis in the C1 subtype. Similarly, the activity level of immune-related pathways such as TGF beta signaling pathway, chemokine signaling pathway, and intestinal network for IgA production, has been proven to be associated with GC treatment and prognosis [56–58], our research results also confirmed significant differences in these pathways between the C1 and C2 subtypes, validating the accuracy of the GC classification model based on parthanatos scores that we constructed.

The tumor immune microenvironment plays a crucial role in the progression of malignant tumors, involving both host anti-tumor immune responses and the destruction of normal tissues. Increasing evidence suggests that immune cell infiltration, such as CD4+ T cells and B lymphocytes, plays a key role in various cancers, including GC [59–61]. However, the potential correlation between the development of GC and immune cell infiltration landscape has not been fully determined. Based on the GC clustering model we established, we attempted to explore the relationship between parthanatos and the immune microenvironment within GC. The ESTIMATE analysis can calculate the proportions of immune cells, stromal cells, and tumor cells within tumor tissues. Our results showed that the C1 subtype had a higher proportion of stromal cells and immune cells in tumor tissues, while the C2 subtype had higher tumor purity. Using the algorithms provided by the TIMER2.0 platform, we found differences in the level of immune cell infiltration between the C1 and C2 subtypes, with a significantly higher number of B cells in the C1 subtype. It has been reported that tumor-infiltrating B cells can regulate the pro-angiogenic effect of bone marrow cells through the secretion of soluble mediators, and they also promote tumor growth by blocking T cell-mediated immune responses through the production of lymphotoxins [62, 63]. This again validates the reason why patients in the C1 subtype have a worse prognosis. Similar differences were found in the expression of immune checkpoint-related genes, such as CD8A, CD44, NRP1, and TNFSF14, which were significantly higher in the C1 subtype. It is well known that immune checkpoints control immune responses, such as effector T cells and NK cells, through various mechanisms [64], when immune checkpoint-related genes are upregulated, the activity of immune cells is suppressed, and more immune cells are recruited into the tumor microenvironment to participate in anti-tumor immune processes under the influence of chemokines and other cytokines [65, 66], which is consistent with the results of our ESTIMATE analysis. Furthermore, using the ssGSEA algorithm, we also analyzed the relationship between parthanatos scores and immune cell populations and functions in the 743 GC samples. We found that different parthanatos-related genes had varying degrees of correlation with various immune cells or functions, and parthanatos scores were also correlated with classic immune cell infiltration, primarily in a positive manner.

Currently, drugs such as sorafenib, rapamycin, vincristine, and MG-132 have been shown to have certain anti-cancer effects in GC. Among them, sorafenib significantly increases the expression of caspase-3, Bax, cyt-c proteins in a dose-dependent manner and reduces the expression of Bcl-2 protein. Inactivation of ERK protein phosphorylation is one of the mechanisms by which sorafenib inhibits GC [67]; rapamycin effectively blocks S1K4, 1E-BP-1, and HIF-31α activation *in vitro* in GC cells, significantly inhibiting tumor cell migration [68]; MG-132, as a ubiquitin-proteasome inhibitor, can significantly inhibit telomerase activity in GC cells, induce cell apoptosis, and cause G1 arrest [69]. Based on the “oncoPredict” package and the GDSC2 dataset, we conducted an analysis on tumor drug sensitivity prediction, predicting the responses of 743 GC patients to various targeted drugs, and analyzed potential beneficial drugs for different parthanatos subtypes of GC patients. Ultimately, we found that patients of subtype C1 were more sensitive to dasatinib, while patients of subtype C2 were more sensitive to carmustine, sorafenib, rapamycin, sepantronium, vincristine, MG-132, lapatinib, epirubicin, osimertinib, cytarabine, and docetaxel. This result will provide precise guidance for the rational use of drugs in GC patients.

Finally, based on the “limma” package, we selected differentially expressed genes between subtypes C1 and C2. We used univariate Cox regression to screen for genes related to GC prognosis, LASSO regression to filter genes, and multivariate Cox regression to build a prognostic model. Finally, we obtained a GC prognostic risk model consisting of 14 parthanatos-related genes (SGCA, JAM2, SHISA3, DES, PDK4, SFRP2, GRP, TNC, PAEP, FBLN5, GLDC, CCDC80, HAND2, and PPP1R14A). By using the predict function, we can calculate the risk score for each GC patient, where higher risk scores often correspond to poorer survival outcomes. Among these genes, a considerable proportion has been proven to be closely associated with GC development. For example, JAM2 belongs to the immunoglobulin superfamily cell adhesion molecules and plays a crucial role in maintaining cell-cell junction integrity. Imbalanced JAM2 gene expression has been correlated with GC staging, differentiation, and progression [70]; the pyruvate dehydrogenase kinase encoded by the PDK4 gene is a crucial enzyme that maintains a high rate of glycolysis in cancer cells, promoting resistance to apoptosis, it has been shown to enhance proliferation and invasion of GC tumor cells and is associated with infiltrations of B cells, CD4+ T cells, and dendritic cells, making it an adverse prognostic factor in GC [71], which aligns with our findings. Similarly, SFRP1, GLDC, HAND2, and other genes have also been found to play critical roles in the development and prognosis of GC [72–75].

To evaluate the modeling performance and predictive accuracy of the model, we compared the prognostic differences between different risk groups in four cohorts, including the train cohort. We plotted ROC curves and calculated the AUC values. The stability of the model was validated through internal and external validation strategies. For the high- and low-risk groups classified by the model, we also conducted comparative analysis of immune infiltration and immune checkpoint-related genes. We found that there were still differences between the two groups, indicating the significant role of the immune microenvironment. Among them, we observed a significantly higher number of cancer-associated fibroblasts (CAFs) in the high-risk group than in the low-risk group, which may be one of the important reasons for the decreased number of immune cells and upregulation of immune checkpoint-related genes in the high-risk group. It has been reported that CAFs and their related biomarkers are associated with poor prognosis in various types of cancer [76, 77], apart from directly promoting tumor growth, metastasis, and angiogenesis, CAFs may also mediate tumor immune escape by directly inhibiting immune cell infiltration and activity or by promoting the recruitment of immunosuppressive cells [78, 79]. In addition, based on this model, we also generated column charts to predict the survival rates of GC patients at 1, 3, and 5 years by integrating risk group, grade, stage, gender, and age data.

Furthermore, our research has certain limitations primarily due to the fact that it only includes bioinformatics analysis. For a detailed understanding of the mechanism of action between parthanatos and GC, further validation from *in vivo* and *in vitro* experiments is needed to support our conclusions. Additionally, finding effective predictive biomarkers for diagnosis and prognosis in malignant tumors is a challenging task for us, and future studies should include larger sample sizes to improve our research findings.

## CONCLUSIONS

Through integrating a series of bioinformatics methods, we explored the potential link between parthanatos and GC. Single-cell combined spatial transcriptomic analysis highlighted the difference in signal expression of parthanatos between cells in GC samples, with almost all cells within tumor tissues and metastatic tissue displaying higher parthanatos signals compared to normal tissues. Based on the expression levels of parthanatos-related genes, we divided GC patients into two subtypes, which showed significant differences in prognosis, immune infiltration, and tumor purity, suggesting a relationship between the development of GC and aberrant parthanatos pathway. We also created and validated a novel prognostic risk model based on parthanatos-related genes, which showed good predictive capability. Higher risk scores were associated with poorer survival outcomes, potentially providing a more targeted and accurate new strategy for the treatment and prognosis of GC patients.

## Supplementary Material

Supplementary Figures
