# Supplementary material for: Integrated single-cell sequencing, spatial transcriptome sequencing and bulk RNA sequencing highlights the molecular characteristics of parthanatos in gastric cancer
Source: Aging (Albany NY). 2024 Mar 18;16(6):5471–500. doi: 10.18632/aging.205658 (PMC11006479; doi:10.18632/aging.205658)
Supplement: Supplementary Figures [file aging-16-205658-s001.pdf]

SUPPLEMENTARY FIGURES

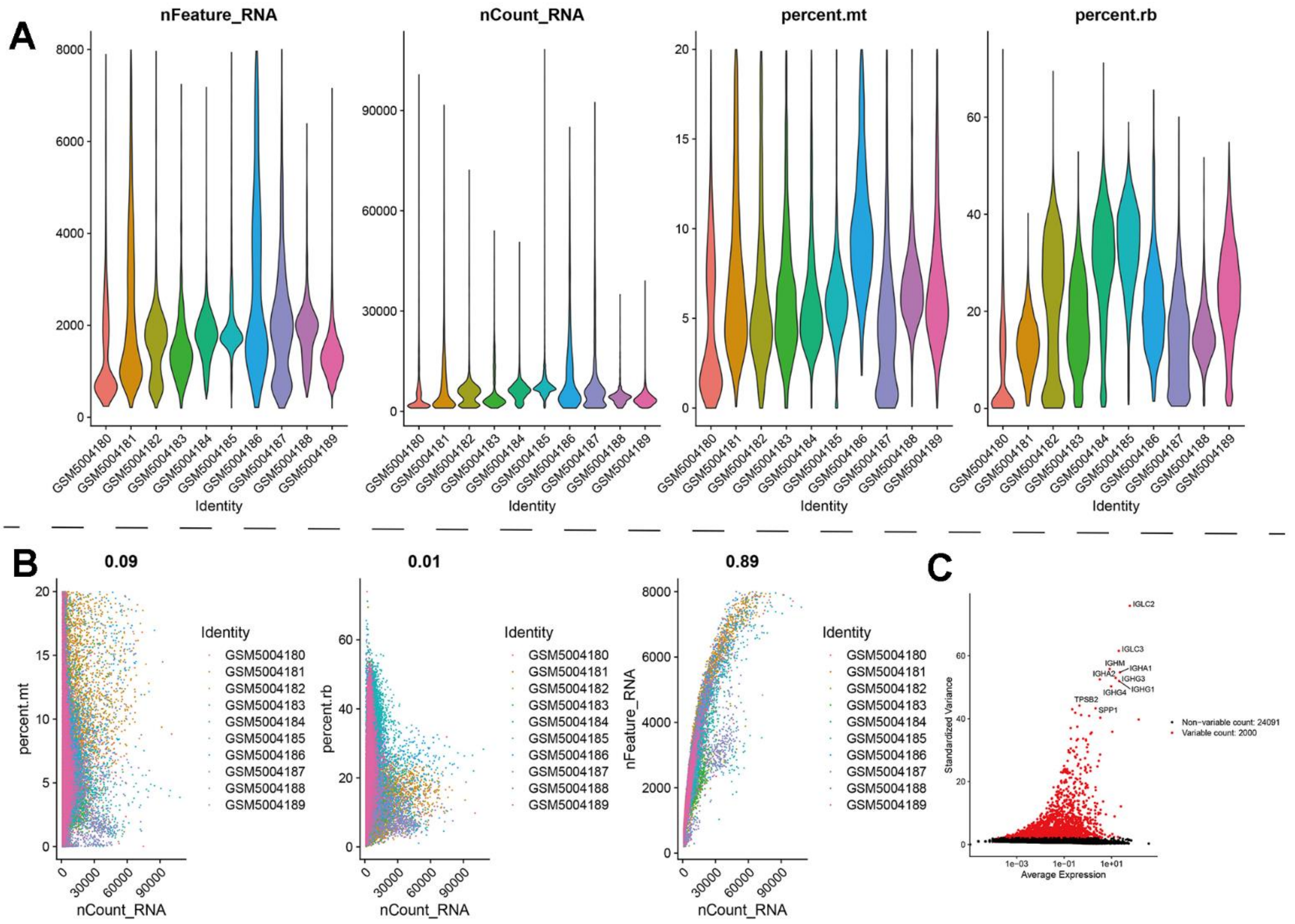

Supplementary Figure 1. Data quality control. By controlling the sequencing depth, number of genes, mitochondrial content and ribosome content, the unqualified cells are filtered out. (**A**, **B**) Unsatisfied cells were filtered based on sequencing depth, number of genes, mitochondrial content, and ribosome content. (**C**) Screen for highly variable genes.

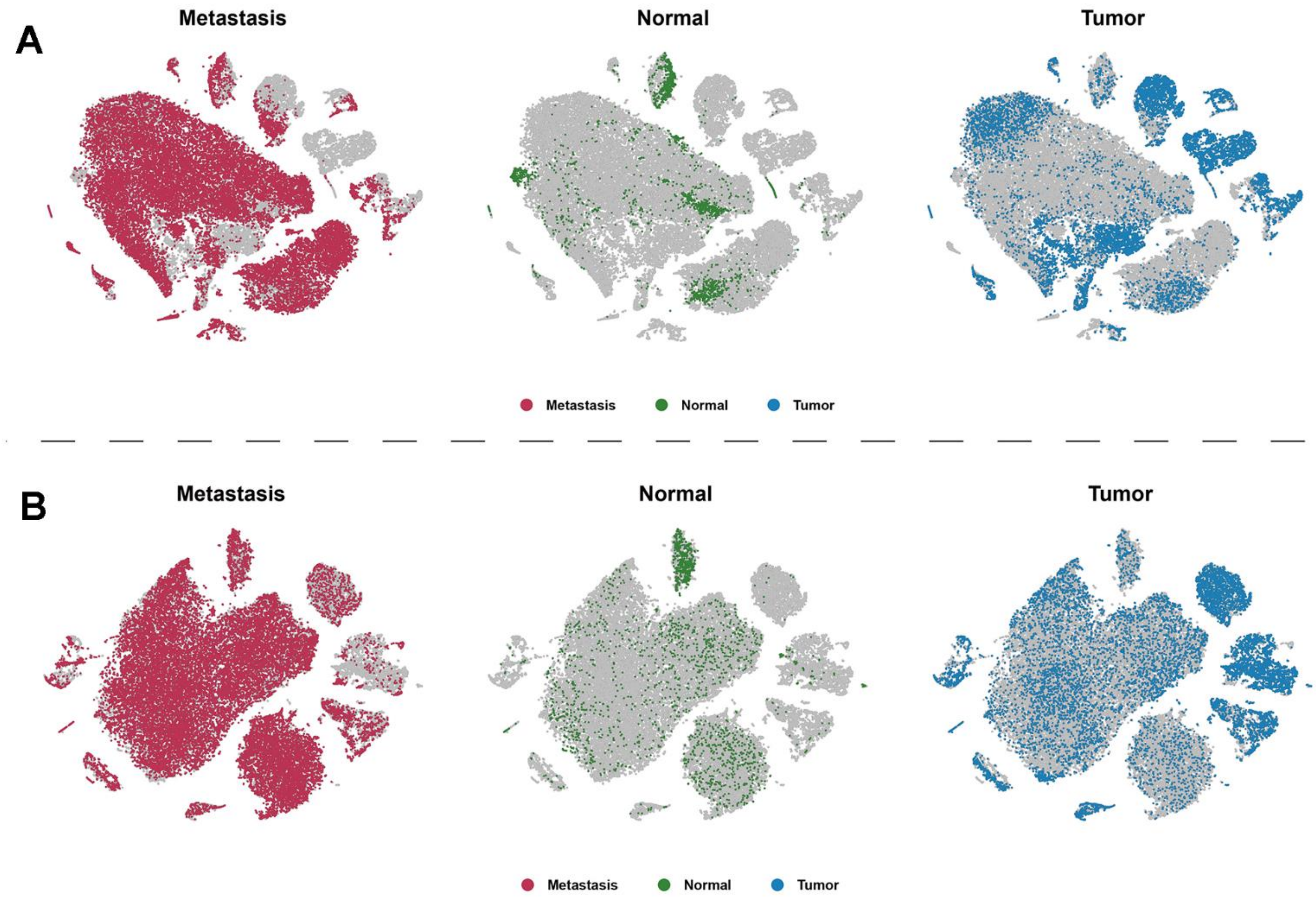

Supplementary Figure 2. UMAP nonlinear clustering algorithm was used to reduce and annotate the combined samples, and the normal samples and tumor samples were separated. (**A**) Distribution of normal samples and tumor samples before dimensionality reduction. (**B**) Distribution of normal samples and tumor samples after dimensionality reduction.

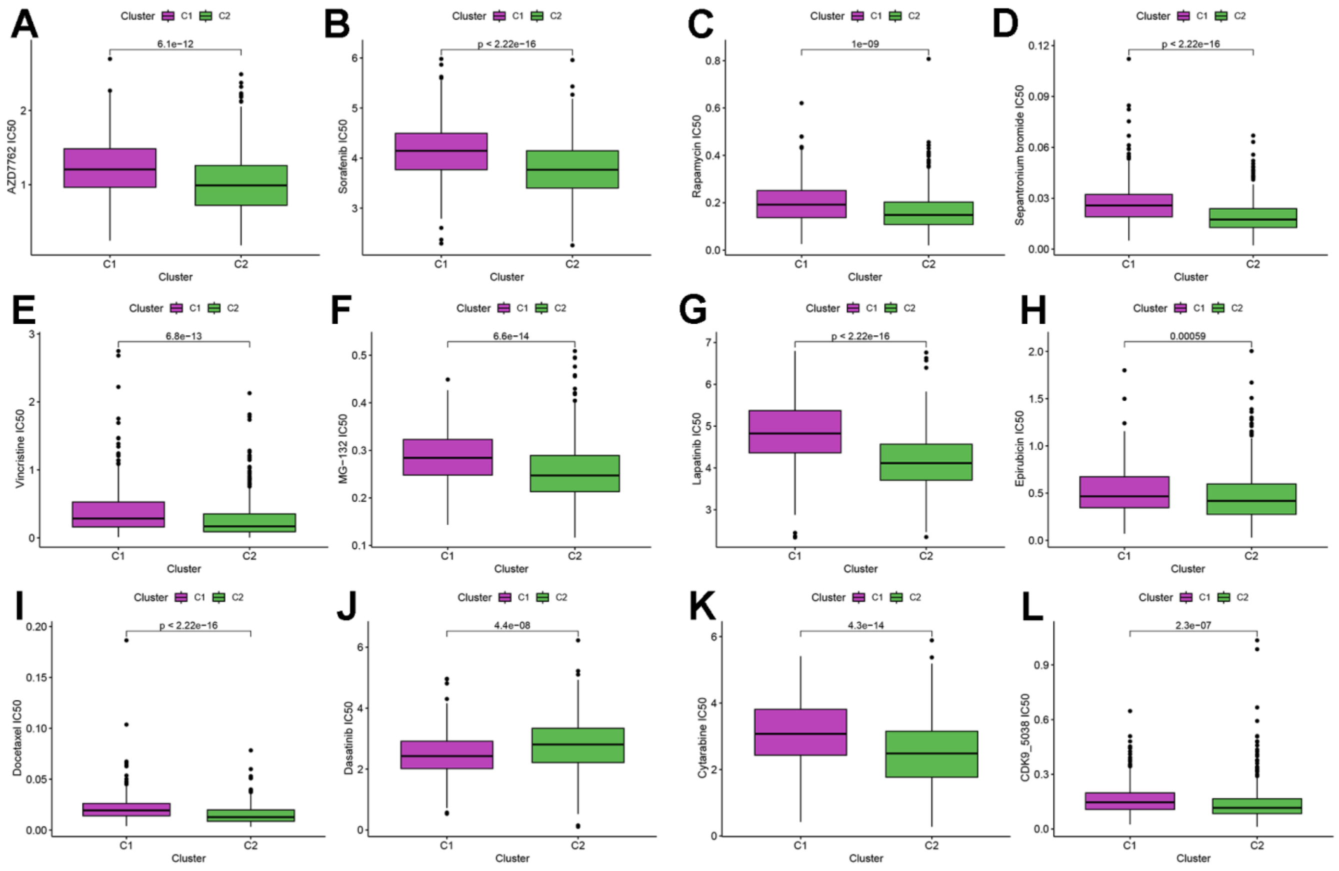

Supplementary Figure 3. Drug sensitivity analyses of CI and C2 subtypes to AZD7762. (**A**), sorafenib (**B**), rapamycin (**C**), sepantronium bromide (**D**), vincristine (**E**), MG-132 (**F**), lapatinib (**G**), epirubicin (**H**), docetaxel (**I**), dasatinib (**J**), cytarabine (**K**), and CDK9-5038 (**L**).

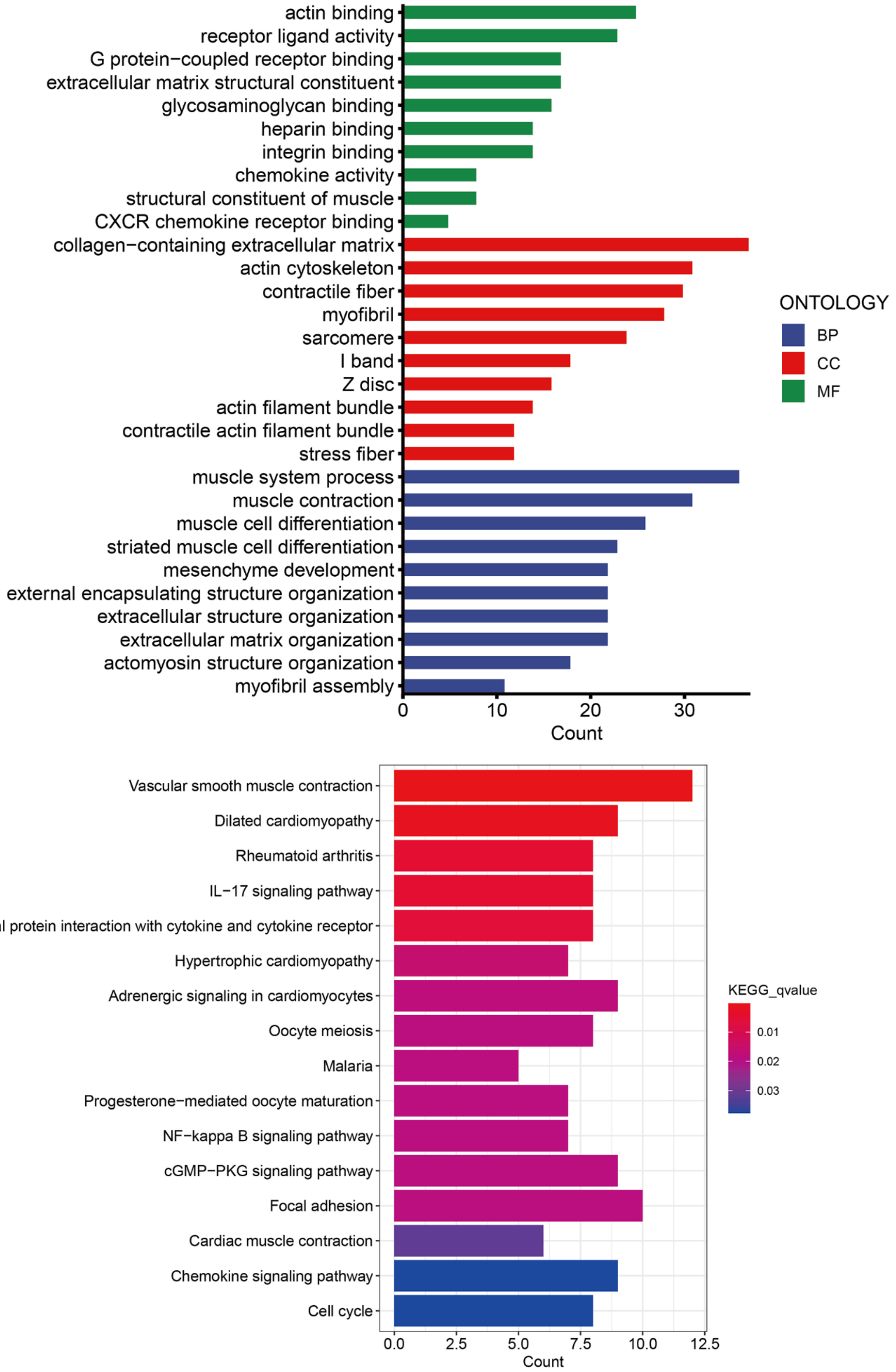

Supplementary Figure 4. GO and KEGG analysis of differentially expressed genes from C1 and C2 subtypes.

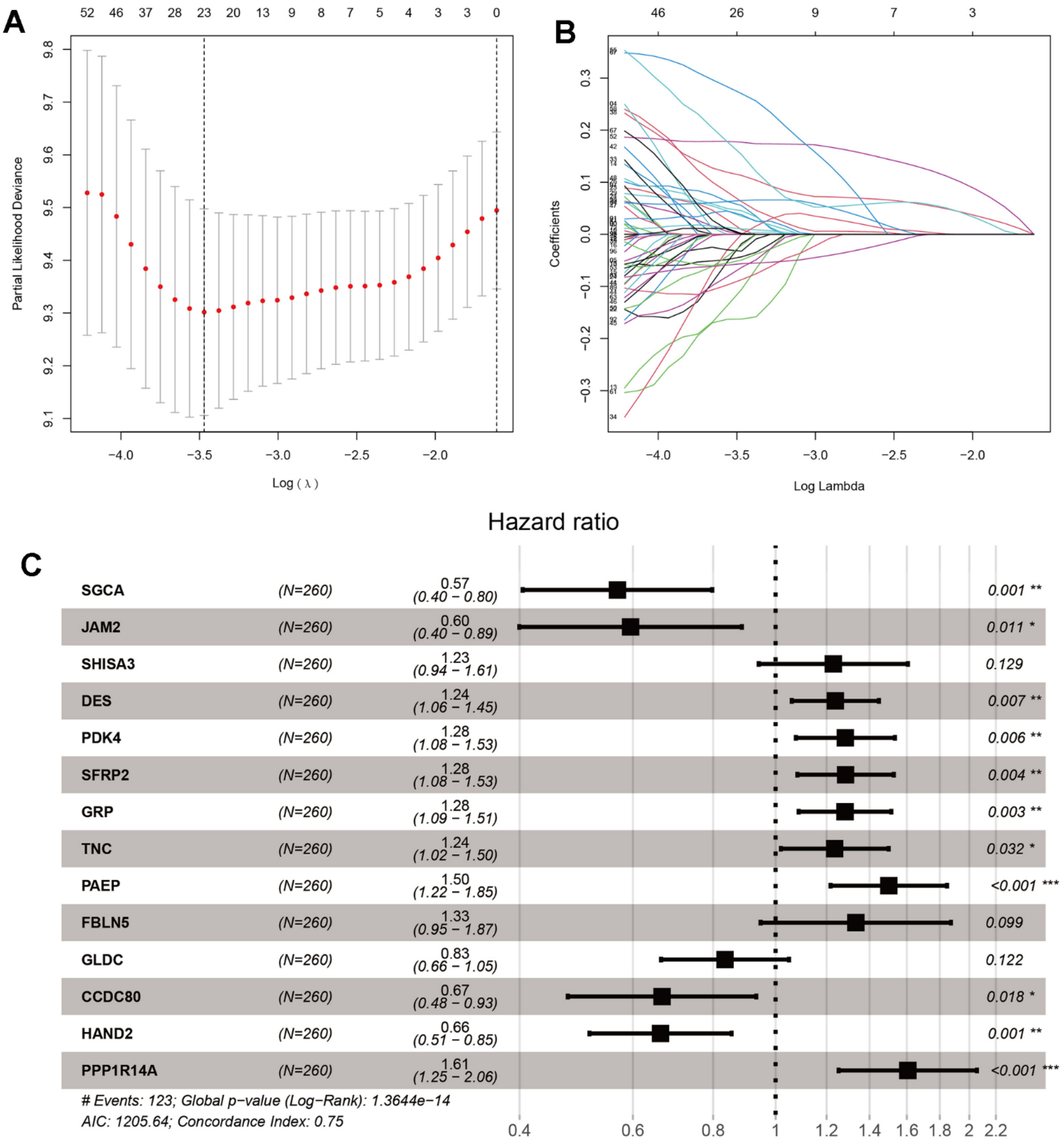

Supplementary Figure 5. LASSO regression analysis and Cox regression analysis were used to construct the prognosis model. (**A**, **B**) LASSO coefficient spectra of prognostic related genes in GC. (**C**) Forest map showing 14 model genes and their hazard ratios.

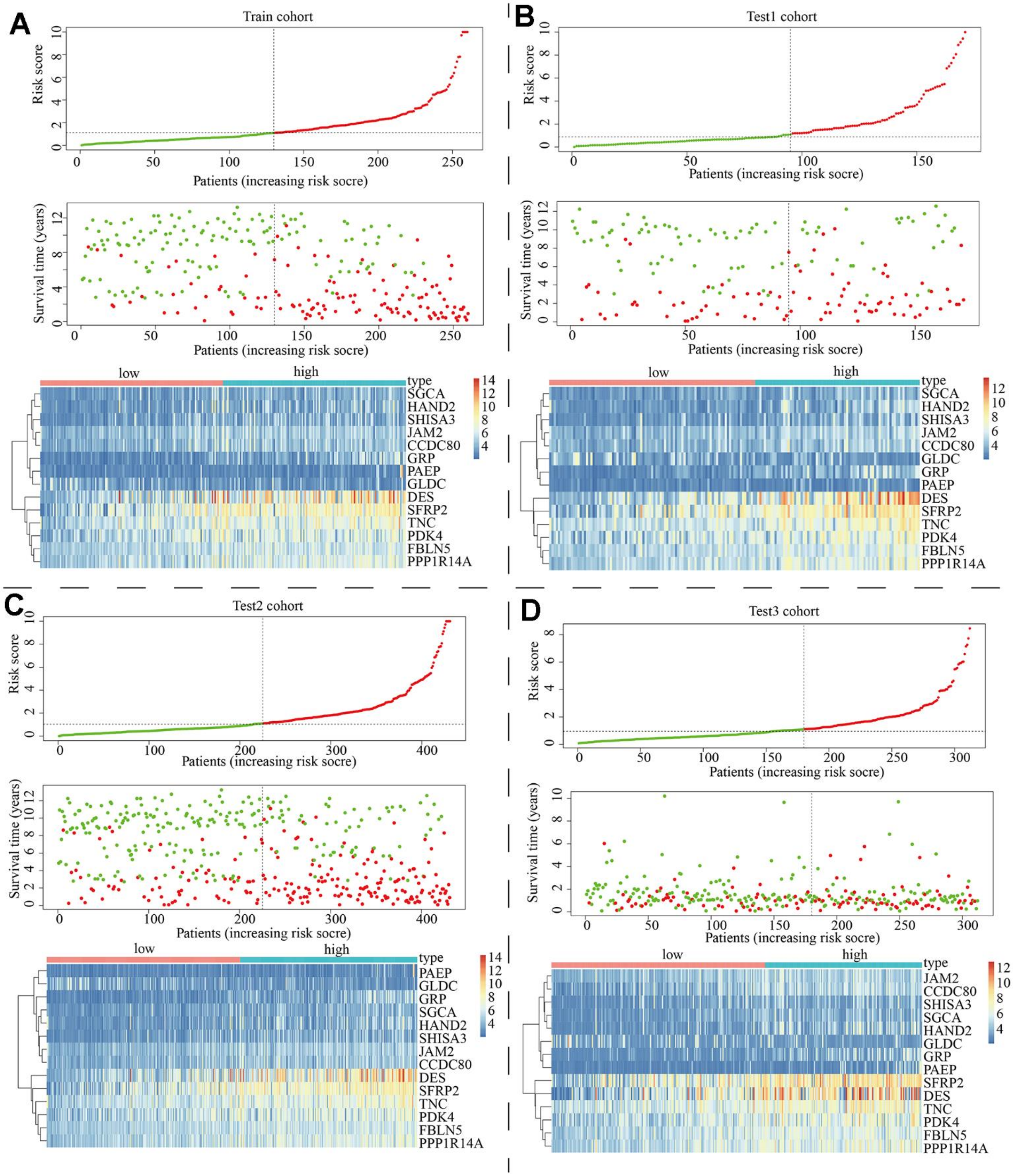

Supplementary Figure 6. Patient partitioning based on median risk score. Survival time distribution of high- and low-risk groups and expression difference of parthanatos-related genes between high- and low-risk groups in train cohort (**A**), test1 cohort (**B**), test2 cohort (**C**) and test3 cohort (**D**).

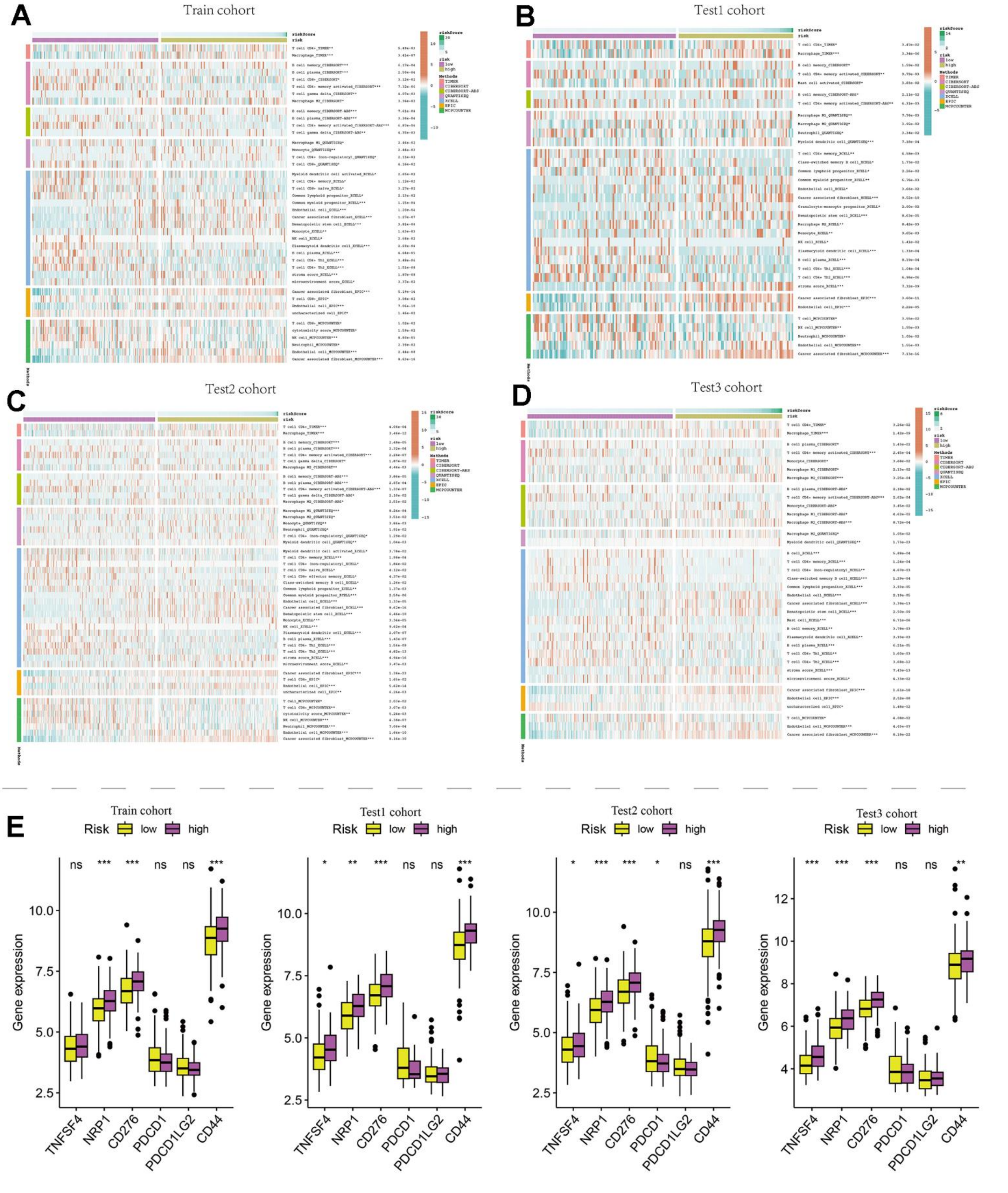

Supplementary Figure 7. Differences in the immune microenvironment between high- and low-risk groups in the train cohort. (**A**), test1 cohort (**B**), test2 cohort (**C**) and test3 cohort (**D**), including seven different algorithms and differences in immune checkpoint related gene expression (**E**).
